# Supplementary material for: Diet-induced metabolic and immune impairments are sex-specifically modulated by soluble TNF signaling in the 5xFAD mouse model of Alzheimer’s disease
Source: bioRxiv. 2024 Feb 28:2024.02.28.582516. Preprint. [Version 1] doi: 10.1101/2024.02.28.582516 (PMC10925304; doi:10.1101/2024.02.28.582516)
Supplement: Supplement 1 — Supplemental Figure S1. High-fat high-carbohydrate impact on metabolic pathways in the hippocampus and cortex of 5xFAD female mice. Densitometric quantification of western blot analysis of hippocampal and cortical tissue from female 5xFAD mice fed 8wks of a CD or HFHC, for the indicated metabolic signaling proteins. Points represent individuals and bars the mean and SEM. Data assessed by unpaired, two-tailed T-test. * p ≤0.05, **p ≤ 0.01, ***p ≤ 0.001, ****p ≤ 0.0001. Supplemental Figure S2. Amyloid beta abundance is not altered by HFHC in female 5xFAD mice. (A-D) Aβ 38, 40 and 42 in soluble homogenates from (A, B) hippocampal and (C, D) cortical tissue quantified by multiplex immunoassays from 5xFAD mice after 8wks of CD or HFHC consumption. Points represent individuals and bars the mean and SEM. Data assessed by unpaired, two-tailed T-test. ns=non-significant. Supplemental Figure S3. High-fat high-carbohydrate intake is associated with intestinal dysregulation. (A) Colon length, (B) small intestine length, and (C) cecum weight of female 5xFAD mice fed 8wks of a CD or HFHC. (D-H) Western blot analysis of proximal colon for occludin, ZO-1, claudin 2, nNOS and PGP9.5. Points represent individuals and bars the mean and standard error. Data assessed by unpaired, two-tailed T-test. **p ≤ 0.01, ***p ≤ 0.001, ****p ≤ 0.0001. Supplemental Figure S4. Diet-induced obesity impact on intestinal microbiome in 5xFAD mice. (A) PCA plots based on Bray-Curtis distance of 16S microbiome profiles from mice receiving the CD or (B) HFHC over 8wks. (C, D) Shannon alpha diversity indices comparing intestinal microbial community between 5xFAD fecal samples from mice fed a CD or HFHC diet at the (C) 4 wks or (D) 8 wks time points post-diet intervention. (E) Genera-level alterations between diets at 8wks, determined by two-tailed T test. Points represent individuals (A, B), and boxes the range (C, D). N=5 from individual cages. [file media-1.pdf]

Supplemental Table 1. Hippocampal tissue from female mice comparing HFHC-diet, Saline-treated to Control diet, Saline treated.

Gene	*p* value	Fold change	Adj *p* value
Grin1	0.0087	-1.5	0.65
Lrrc25	0.039	-1.9	0.66
Shh	0.0098	-1.6	0.65
Cldn5	0.00051	1.7	0.38
Dll4	0.006	1.7	0.55
Flt4	0.017	2.1	0.65
Ggt1	0.018	1.58	0.65
Hspb1	0.013	1.8	0.65
Il1b	0.023	2.3	0.65
Itpr3	0.0031	3.3	0.47
Jun	0.024	1.5	0.65
Kel	0.041	1.85	0.66
Mmrn2	0.017	2.07	0.65
Mpz	0.024	2.8	0.65
Mthfr	0.028	2.1	0.66
Myd88	0.048	1.5	0.66
Pla2g2e	0.017	1.9	0.65
Prl	0.016	1.87	0.65
Sh3tc2	0.0046	2	0.54
Slc6a4	0.0015	2.9	0.38
Tlr2	0.019	1.7	0.65
Tnfrsf10b	0.027	1.7	0.66
Uchl1	0.013	1.7	0.65

Supplemental Table 2. Hippocampal tissue from female mice comparing HFHC-diet, XPro-treated to HFHC-diet, Saline treated.

Gene	*p* value	Fold change	Adj *p* value
Tnc	0.038	-2.16	0.97
ADRB2	0.038	1.7	0.97
C3	0.003	2.51	0.97
Chat	0.03	3.84	0.97
Egr2	0.013	2.19	0.97
Fas	0.049	1.6	0.97
Fos	0.0066	1.84	0.97
Grin3b	0.0065	2.72	0.97
Marco	0.015	2.85	0.97
Mutyh	0.047	2.31	0.97
Plcb4	0.0099	1.94	0.97
Slc18a3	0.032	3.16	0.97
Smyd1	0.0019	2.55	0.97

Supplemental Table 3. Hippocampal tissue from male mice comparing HFHC-diet, Saline-treated to Control diet, Saline treated.

Gene	*p* value	Fold change	Adj *p* value
Cybb	0.0018	1.56	0.56
Fas	0.02	1.82	0.86
Gabrr3	0.034	3.3	0.93
Gpr84	0.03	1.51	0.93
Icam1	0.014	2.68	0.79
Mmp19	0.0093	3.26	0.71
Tlr4	0.0031	1.91	0.56

Supplemental Table 4. Hippocampal tissue from male mice comparing HFHC-diet, XPro-treated to HFHC-diet, Saline treated.

Gene	*p* value	Fold change	Adj *p* value
Ager	0.037	-2.18	0.97
Csf2rb	0.034	-2.62	0.97
Fas	0.05	-1.69	0.97
Gabrr3	0.021	-3.21	0.97
Mmp19	0.016	-3.3	0.97
Nkx6-2	0.018	-2.36	0.97
Phf19	0.0016	-2.89	0.58
Tnfrsf12a	0.0058	-1.53	0.88
Pla2g4e	0.042	2.63	0.97

Supplemental Table 5. Frontal cortex tissue from female mice comparing HFHC-diet, Saline-treated to Control diet, Saline treated.

Gene	*p* value	Fold change	Adj *p* value
Casp7	0.048	-1.8	0.98
Klk6	0.031	-2	0.98
Pla2g4c	0.04	2.2	0.98
Ret	0.011	2.3	0.98
Tlr4	0.0066	2.6	0.98

Supplemental Table 6. Frontal cortex tissue from female mice comparing HFHC-diet, XPro-treated to HFHC-diet, Saline treated.

Gene	*p* value	Fold change	Adj *p* value
Adora2a	0.022	-4.69	1
Cacna1s	0.04	-2.18	1
Chat	0.032	-2.85	1
Drd2	0.0071	-3.71	1
Itga5	0.024	-2.12	1
Pde1b	0.03	-1.61	1
Hmox1	0.0093	1.54	1
Ngf	0.00077	1.76	0.59
Tlr2	0.031	1.67	1

Supplemental Table 7. Frontal cortex tissue from male mice comparing HFHC-diet, Saline-treated to Control diet, Saline treated.

Gene	*p* value	Fold change	Adj *p* value
Mmp12	0.023	-2.79	0.56
Tnfrsf10b	0.009	-3.04	0.39
Ang	0.014	1.38	0.43
C1qb	0.0024	1.31	0.38
C1qc	0.0076	1.25	0.39
Cd44	0.03	1.34	0.67
Cd68	0.042	1.29	0.78
Gfap	0.00061	1.38	0.23
Gusb	0.004	1.29	0.38
Mmp19	0.0084	3.32	0.39
Stab1	0.0028	1.35	0.38
Tcirg1	0.044	1.31	0.79
Trem2	0.0079	1.42	0.39

Supplemental Table 8. Frontal cortex tissue from female mice comparing HFHC-diet, XPro-treated to HFHC-diet, Saline treated.

Gene	*p* value	Fold change	Adj *p* value
Cd14	0.0067	-1.51	1
Lrrc25	0.038	-2.76	1
Plcb2	0.03	-1.59	1
Smyd1	0.041	-2.75	1
