# Supplementary figures and images for: Diet-induced metabolic and immune impairments are sex-specifically modulated by soluble TNF signaling in the 5xFAD mouse model of Alzheimer’s disease
Source: bioRxiv. 2024 Feb 28:2024.02.28.582516. Preprint. [Version 1] doi: 10.1101/2024.02.28.582516 (PMC10925304; doi:10.1101/2024.02.28.582516)

# Supplemental Figure S1

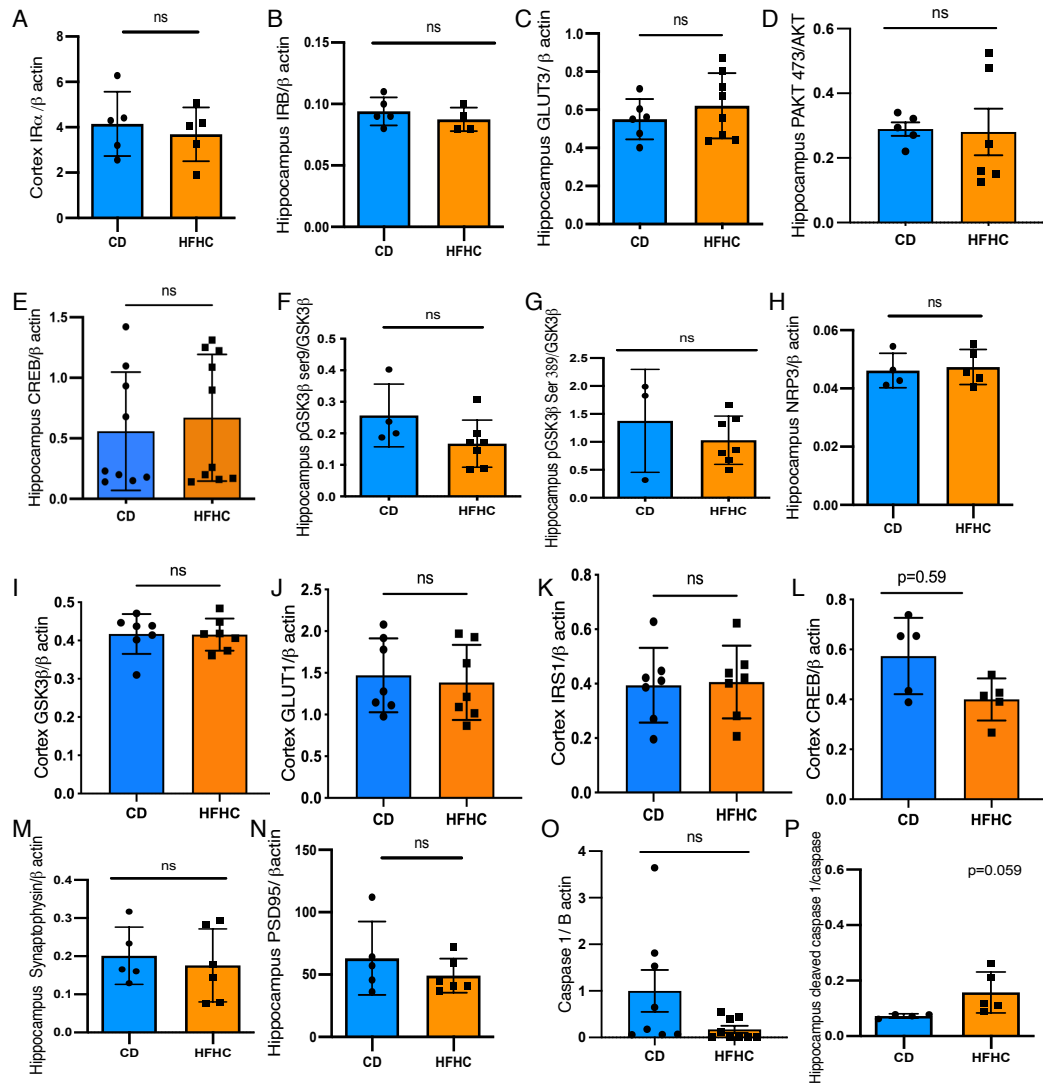

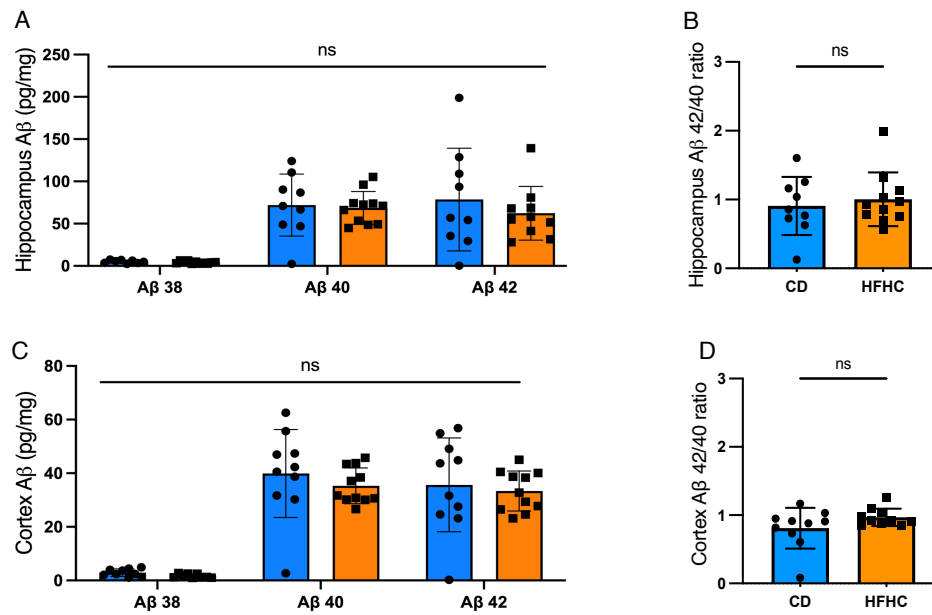

# Supplemental Figure S3

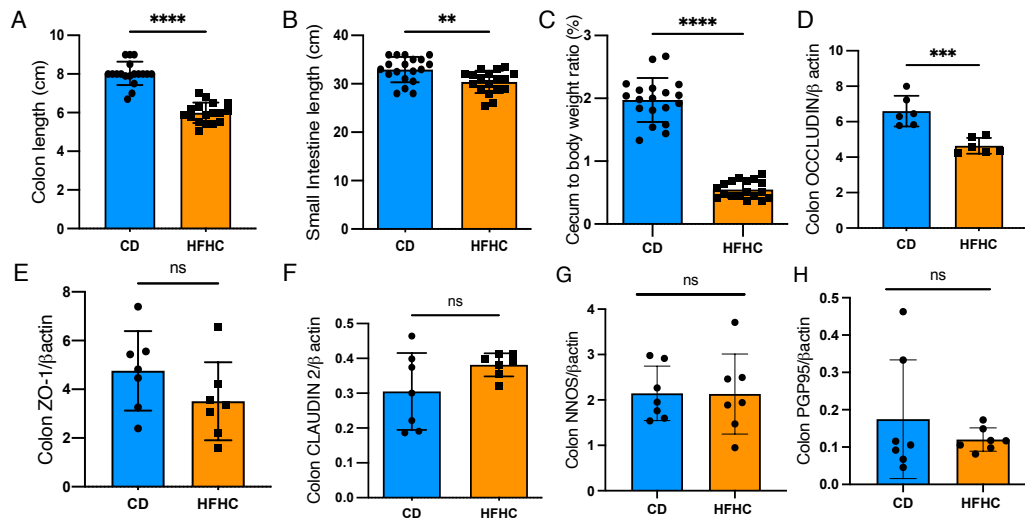

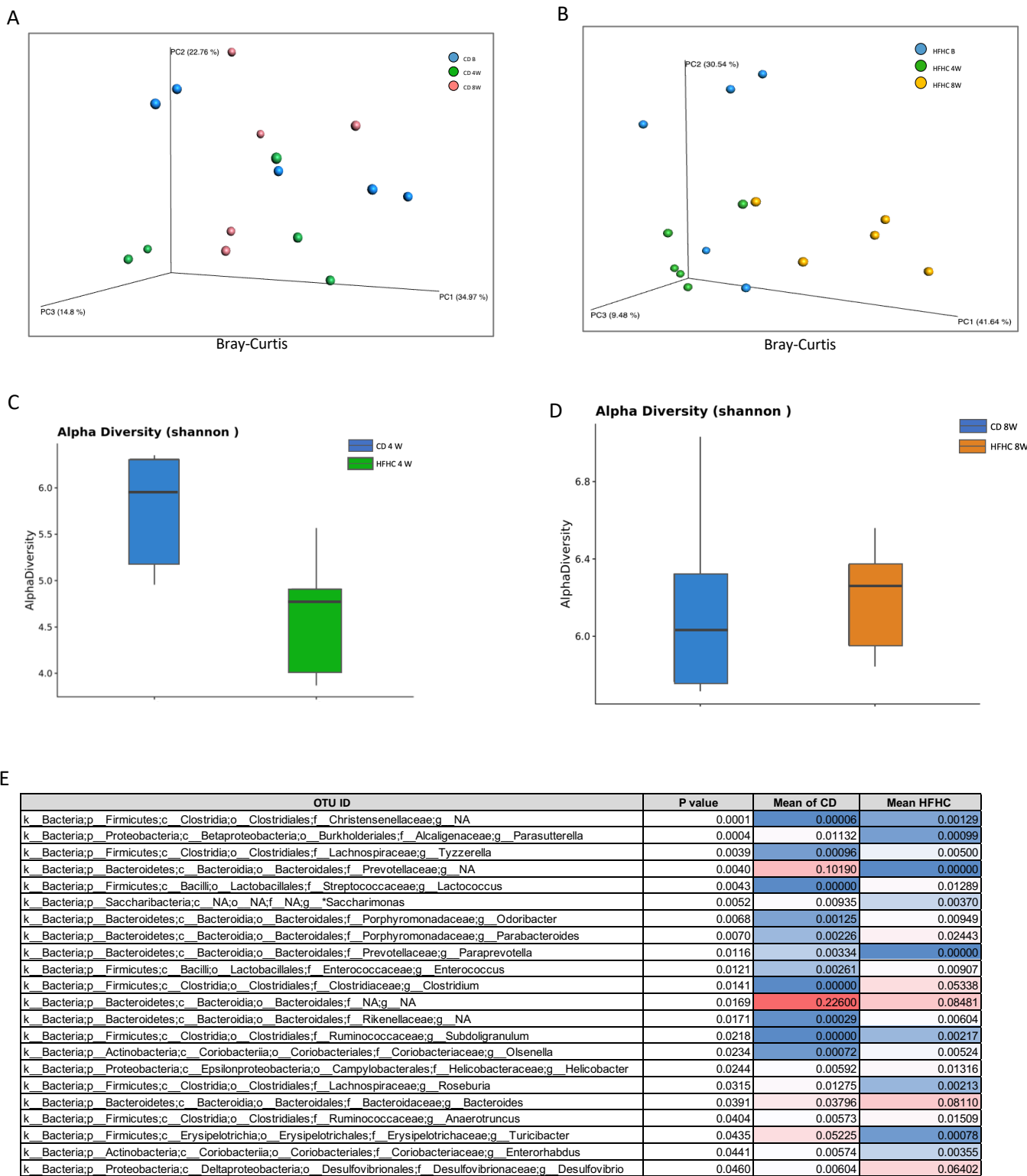

Supplement: Supplement 2 [file NIHPP2024.02.28.582516v1-supplement-2.pdf]
